# Comparison of the microbiome, metabolome, and lipidome of obese and non-obese horses

**DOI:** 10.1371/journal.pone.0215918

**Published:** 2019-04-23

**Authors:** Michelle C. Coleman, Canaan M. Whitfield-Cargile, Rodolfo G. Madrigal, Noah D. Cohen

**Affiliations:** Department of Large Animal Clinical Sciences, Texas A&M University, College Station, TX, United States of America; Wageningen University, NETHERLANDS

## Abstract

Metabolic diseases such as obesity and type 2 diabetes in humans have been linked to alterations in the gastrointestinal microbiota and metabolome. Knowledge of these associations has improved our understanding of the pathophysiology of these diseases and guided development of diagnostic biomarkers and therapeutic interventions. The cellular and molecular pathophysiology of equine metabolic syndrome (EMS) and obesity in horses, however, remain ill-defined. Thus, the objectives of this study were to characterize the fecal microbiome, fecal metabolome, and circulating lipidome in obese and non-obese horses. The fecal microbiota, fecal metabolome, and serum lipidome were evaluated in obese (case) horses (n = 20) and non-obese (control) horses (n = 20) matched by farm of origin (n = 7). Significant differences in metabolites of the mitochondrial tricarboxylic acid cycle and circulating free fatty acids were identified in the obese horses compared to the non-obese horses. These results indicate that the host and bacterial metabolism should be considered important in obese horses. Further studies to determine whether these associations are causal and the mechanistic basis of the association are warranted because they might reveal diagnostic biomarkers and therapeutic interventions to mitigate obesity, EMS, and sequelae including laminitis.

## Introduction

Obesity is of rising concern for the health and well-being of the horse, with a reported prevalence of 31% in the United Kingdom [1]. An obese body condition score (BCS) is associated with an increased risk of hyperinsulinemia [2–4], insulin dysregulation (ID) [5], and laminitis [6]. The combination of obesity, ID, and laminitis are components of equine metabolic syndrome (EMS) [7]. Our current understanding of EMS is based on diagnostic tests that inadequately assess the pathophysiology of the condition at the cellular and molecular levels. Consequently, our ability to diagnose, manage, and prevent this syndrome is limited.

The gastrointestinal microbiota has been shown to be a crucial factor in the development of metabolic diseases such as obesity and type 2 diabetes in people. This is thought to be a result of the microbiota’s modulation of host energy metabolism [8], gastrointestinal epithelial permeability [9], regulation of gastrointestinal peptide hormone secretion [10], and host inflammation [10, 11]. The metabolic relationship between the host and the gastrointestinal microbiota has encouraged metabolomic approaches to identifying key mechanisms linking bacterial composition with host responses that contribute to the development of disease. New techniques in metabolomic analysis have allowed for comprehensive quantification of metabolic intermediates from biological systems, and have proven to be a powerful tool for biomarker discovery in human medicine [12]. Specifically, altered products of the microbial and host metabolism of bile acids [13, 14], vitamins [15], branched chain fatty acids [16, 17], and purines and phenolic compounds [13] have been identified in people with metabolic disease relative to healthy controls. This knowledge has led to the development of tools for early diagnosis [18, 19] and monitoring response to therapy [20].

The gastrointestinal microbiota of horses is naturally highly diverse and variable in composition [21–23]. As hind-gut fermenters, the intestinal microbiota plays a critical role in cellulose fermentation and energy production in horses. Although the microbial composition of the microbiota is important, the metabolites of these organisms are a major mechanism influencing the health of the host. For example, production of short-chain fatty acids (SCFA) from the microbial digestion of cellulose provides a substantial portion of the daily energy requirements of a horse. While SCFAs are well-recognized for their effects on host mucosal health and maintenance of mucosal homeostasis, they represent a small fraction of the thousands of metabolites produced by the microbiota each day, many of which also have important impact on host health [24]. Despite the importance of the intestinal microbiota for equine health and availability of molecular techniques for assessing this complex microbial community, information regarding the quantification, characterization, and metabolic activity of the equine gastrointestinal microbiota is limited. Furthermore, to our knowledge, neither the fecal microbiota nor the fecal metabolome have been comprehensively evaluated in obese horses.

There are 4 types of biologic molecules (*viz*., nucleic acids, amino acids, carbohydrates, and lipids). The diversity of lipids is vast, with over 600 distinct molecular species in people [25]. In view of the importance of lipids to biological processes, detailed knowledge of the composition and concentration of lipid metabolites have greatly expanded our understanding of the pathophysiology of disease and improved diagnostic and therapeutic monitoring capabilities [26]. Although horses with EMS have abnormal lipidomes compared to non-EMS control horses [27], the lipidome has not been evaluated comprehensively in the context of equine obesity; not all obese horses have EMS and not all EMS horses are obese. Consequently, an improved understanding of the pathophysiology of obesity in horses holds potential to improve our ability to identify horses at risk for developing obesity. Earlier identification of at-risk horses could lead to targets for interventions to prevent obesity and for biomarkers to identify at-risk horses for which such interventions could be implemented and evaluated. Thus, the objectives of this study were to compare the composition of the fecal microbiota, fecal metabolome, and serum lipidome of obese and non-obese horses.

## Materials and methods

### Study population

Local horse operations in central Texas were recruited through personal communications with the investigators to participate in the study. Farms with horses of obese (case) horses and non-obese (control) horses, defined on the basis of body condition score (BCS), that provided written informed consent for participation were included. A questionnaire was used to capture the following data for each included horse: 1) signalment (age; breed; sex); 2) dietary and feeding practices; and, 3) BCS from 1 (emaciated) to 9 (obese) based on the Henneke scale [28] as determined by a single, veterinary observer (MCC). Cases of obesity were defined as horses having a BCS ≥7 at the time of examination. For each obese horse, an apparently healthy horse with a BCS of ≥ 3- ≤ 5 residing at the same premises as the obese horse was included. Horses were excluded from the study if they had received medications, including vaccinations and anthelmintics, within 30 days of sample collection, had a history of laminitis, or had a current illness. Feces were collected from all horses via rectal palpation. Blood was collected via jugular venipuncture. Fecal and serum samples were frozen at -80°C on the day of collection. This study was approved by the Institutional Animal Care and Use Committee (IACUC 2015–0156) and the Clinical Research and Review Committee of the College of Veterinary Medicine & Biomedical Sciences at Texas A&M University.

### Microbiota

#### Sample processing

Genomic DNA was extracted using a commercially available kit (QIAamp Fast DNA Stool Mini Kit, Qiagen) as previously described with slight modifications to the manufacturer’s protocol [29]. Briefly, 200 mg of frozen feces was placed in a 2-mL tube containing 1 mL Inhibitex buffer and 50 mg each of sterile/DNAase free 0.1- and 0.5-mm silica zirconium beads. The sample was homogenized for 90 seconds at 6.5 m/sec with FastPrep FP120 cell disrupter (Qbiogene, Carlsbad, CA). The sample was heated at 70° C for 10 minutes prior to DNA extraction per the manufacturer’s protocol. DNA was suspended in tris-EDTA buffer and stored at -80°C. Amplification and sequencing of the V4 variable region 16S rRNA gene was performed by a commercial laboratory (www.mrdnalab.com, Shallowater, TX, USA). Briefly, samples were amplified using barcode-tagged PCR primers in a 28-cycle PCR using HotStarTaq Plus Master Mix Kit (Qiagen, USA). The following conditions were used for PCR: 94° C for 3 minutes, followed by 28 cycles at 94° C for 30 seconds, 53° C for 40 seconds, 72° C for 1 minute, and finally a hold at 72° C for 5 minutes. A DNA library was prepared according to Illumina TruSeq DNA library preparation protocol. Sequencing was performed on an Illumina MiSeq and data were uploaded into the NCBI GenBank database with submission number SUB5111094.

#### Data analysis

Descriptive data were generated for case and control horses. The data was assessed for normality using the Shapiro-Wilk method. Normally distributed data were reported as mean ± SD and analyzed using an ANOVA using JMP Pro 14. A P< 0.05 was considered statistically significant.

The software Quantitative Insights Into Microbial Ecology-2 (QIIME2; https://qiime2.org) was used for sequence processing and analysis. The raw sequence data were de-multiplexed, and low quality reads were filtered using database’s default parameters. Sequences were assigned to operational taxonomic units (OTUs) using dada2 against the Greengenes database (ver. gg_13_8) filtered at 97% identity for 16S rRNA sequences. Count tables with assigned taxonomy and phylogenetic trees constructed in QIIME2 were exported to R (ver. 3.5.0) where phyloseq (ver 1.24.2), a software package that utilizes raw (*i*.*e*., unrarefied) data which preserves data from all samples thus retaining data even from samples with fewer reads, was used for further analysis.[30, 31] Alpha diversity was calculated in phyloseq and analyzed using generalized linear models. Beta-diversity was also calculated in phyloseq (via the dependent package vegan [ver 3.5.2]) and analyzed using visual assessment of principal coordinate analysis (PCoA) plots and by analysis of similarity (ANOSIM) [32] calculated on both phylogenetic (e.g., UniFrac) and non-phylogenetic distance metrics (e.g., Bray Curtis) [33]. Differentially expressed OTUs were determined using EdgeR based on the matrix of OTU counts and were not rarefied to an even sampling depth, but instead normalized using EdgeR function [calcnormfactor*s*] with method RLE (relative log expression) [34–36]. An OTU was considered differentially expressed if the false discovery rate (FDR) value of P was < 0.05 [37, 38].

### Fecal metabolomics

#### Sample handling and processing

Fecal and serum samples were maintained at -80°C until metabolic profiling was performed. Global non-targeted mass spectrometry metabolomics analysis was performed at Metabolon, Inc (Metabolon, Inc, Durham, NC), a commercial supplier of metabolic analysis, which has developed a platform that integrates chemical analysis (including identification and relative quantification), data-reduction, and quality assurance. To maximize compound detection and accuracy, 3 separate analytical methods were utilized including ultra-high performance liquid chromatography-tandem mass spectrometry (UHPLC-LC-MS) in both positive and negative ion modes and gas chromatography/mass spectrometry (GC-MS) [39, 40]. Following receipt, samples were inventoried, accessioned into the Metabolon Laboratory Information Management System (LIMS), assigned a unique identifier, and stored at -80°C until analyzed.

Sample preparation was performed by the automated Mircolab STAR system (Hamilton Company, Salt Lake City, UT, USA). Extraction solvents (*i*.*e*., methanol containing recovery standards) were added to each sample. Extraction was carried out by shaking using a Geno/Grinder 2000 (Glen Mills, Clifton, NJ) followed by centrifugation. The resulting extract was divided into 5 fractions: 2 for analysis by 2 separate reverse phase (RP) LC/MS methods with positive ion mode electrospray ionization (ESI), 1 for analysis by RP LC/MS with negative ion mode ESI, 1 for GC/MC analysis, and 1 reserve aliquot. Samples were placed on a TurboVap (Zymark, Midland, Ontario, Canada) to remove organic solvent and were stored overnight under nitrogen before preparation for analysis.

All UPLC-MS/MS methods utilized a Waters Acquity UPLC (Walters, Milford, MA, USA) and a Q-Exactive high resolution/accurate mass spectrometer interfaced with heated electrospray ionization (HESI-II) source and Orbitrap mass analyzer operated at 35,000 mass resolution (Thermo Scientific, Walthram, MA, USA). Sample extracts were reconstituted in solvents compatible to each method employed. The MS analysis alternated between MS and data-dependent MS^n^ scans using dynamic exclusion. The scan range varied slighted between methods but covered mass to charge ratios of 70 to1000 *m/z*. Raw data were archived and extracted. For GC/MS, derivatized samples were separated on a 5% phenyldimethyl silicone column with helium carrier gas. Data were analyzed on a Thermo-Finnigan Trace DSQ MS (Thermo Fisher) operated at a unit mass resolving power with electron impact ionization.

#### Data extraction and compound identification

Detection, identification, integration, and clustering of all ion features into individual compounds was performed using a library of conserved chemical standards using a software developed by Metabolon [41]. Authenticated standards within the library contain retention time/index (RI), mass to charge ratio (*m/z)*, and chromatographic data (including MS/MS spectral data). At present, 3,000 commercially available purified standards are registered into the LIMS for distribution to both the LC and GC platforms for determination of their analytical characteristics. Compound abundance was quantified by calculation of area under the curve for the quantification ion of the compound. Quality control measurements were used to individually check all compounds.

#### Data analysis

To aid data visualization, the raw area counts for each biochemical were rescaled by dividing each sample value by the median for that specific biochemical. For statistical analyses, missing values were assumed to be below the limits of detection and values were imputed with the minimum observed value for each compound. Statistical analysis of log_10_-transformed data were performed. Principal component analysis (PCA) was performed to identify differences across groups. A mixed-model ANOVA was used to identify biochemical that differed significantly between groups, with group as a fixed effect and farm as a random effect to account for clustering by farm. An estimate of false discovery rate (FDR) using *q*-value was calculated to account for multiplicity of comparisons (q value <0.10) [42]. All analyses were performed in Array Studio with a significance set at P<0.05 Raw data are available at 10.6084/m9.figshare.7579778.

### Complex lipid platform

#### Sample handling and processing

Lipids were extracted from serum samples in methanol:dicholoromethane in the presence of internal standards by Metabolon, Inc. The extracts were concentrated under nitrogen and reconstituted in 0.25 mL of 10 mM ammonium acetate dichloromethane:methanol (50:50). The extracts were transferred to inserts and placed in vials for infusion-MS analysis, performed on a Shimazdu LC with nano PEEK tubing and the Sciex Selexlon-5500 QTRAP. Samples were analyzed via both positive and negative mode electrspray. The 5500 QTRAP scan was performed in MRM mode, with more than 1,100 MRMs.

#### Data analysis

Individual lipid species were quantified by taking peak area ratios of target compounds and assigned internal standards, and multiplying them by the concentration of the internal standard added to the sample. Lipid class concentrations were calculated from the sum of all molecular species within a class, and a fatty acid composition was determined by calculating the proportion of each class comprised by individual fatty acids. Data were analyzed in similar fashion as described for the fecal metabolites. Briefly, values were log_10_-transformed with imputation of missing values. A mixed-model ANOVA was used to identify biochemical that differed significantly between obese and non-obese horses, as described above. Data are available at 10.6084/m9.figshare.7579790

## Results

Forty horses (20 obese and 20 non-obese) from 7 farms were included in the study (S1 Table). Of the 40 horses, 31 were geldings and 9 were mares. The mean BCS for obese horses was 7.6 (SD 0.4) and the mean BCS for non-obese horses was 4.6 (SD 0.6). Horses ranged in age from 5 to 24 years, with a mean of 14 years (SD 5.17). There was no significant difference in age between the groups (P = 0.69). Several breeds were represented including Quarter Horses (27), Thoroughbreds (3), Warmbloods (5), Drafts (4), and Arabian (1). All horses were used for pleasure riding.

### Microbiota

Fecal sample reads had a mean of 50,010 reads per sample (range of 29,620 to 76,135) representing 9,372 unique OTUs.

**Fig 1:**
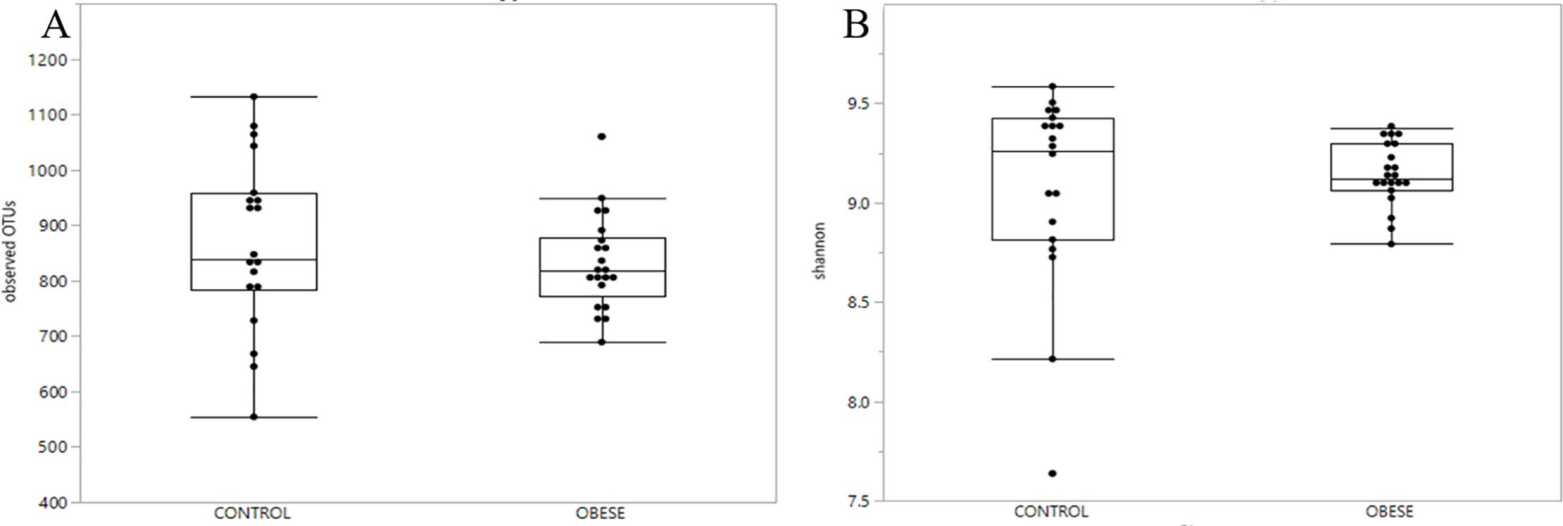
No differences in fecal microbiota alpha richness and diversity among groups. A) Boxplots of observed OTUs for control (non-obese) and obese horses. The horizontal line is the median, and the top and bottom of the box extend to the 75% and 25% percentiles, respectively. The horizontal lines extending outside the box represent multiples of 1.75 times their respective interquartile range There was no statistical difference between groups (P = 0.34). B) Boxplot of Shannon diversity index for control (non-obese) and obese horses. The horizontal line within the box is the median, and the top and bottom of the box extend to the 75% and 25% percentiles, respectively. The horizontal lines extending outside the box represent multiples of 1.75 times their respective interquartile range. There was no statistical difference between groups (P = 0.62).

Alpha diversity and richness index based on the number of observed OTUs and Shannon diversity index, respectively, revealed no significant difference between groups (P = 0.64 and P = 0.32) (Fig 1A and 1B).

**Fig 2:**
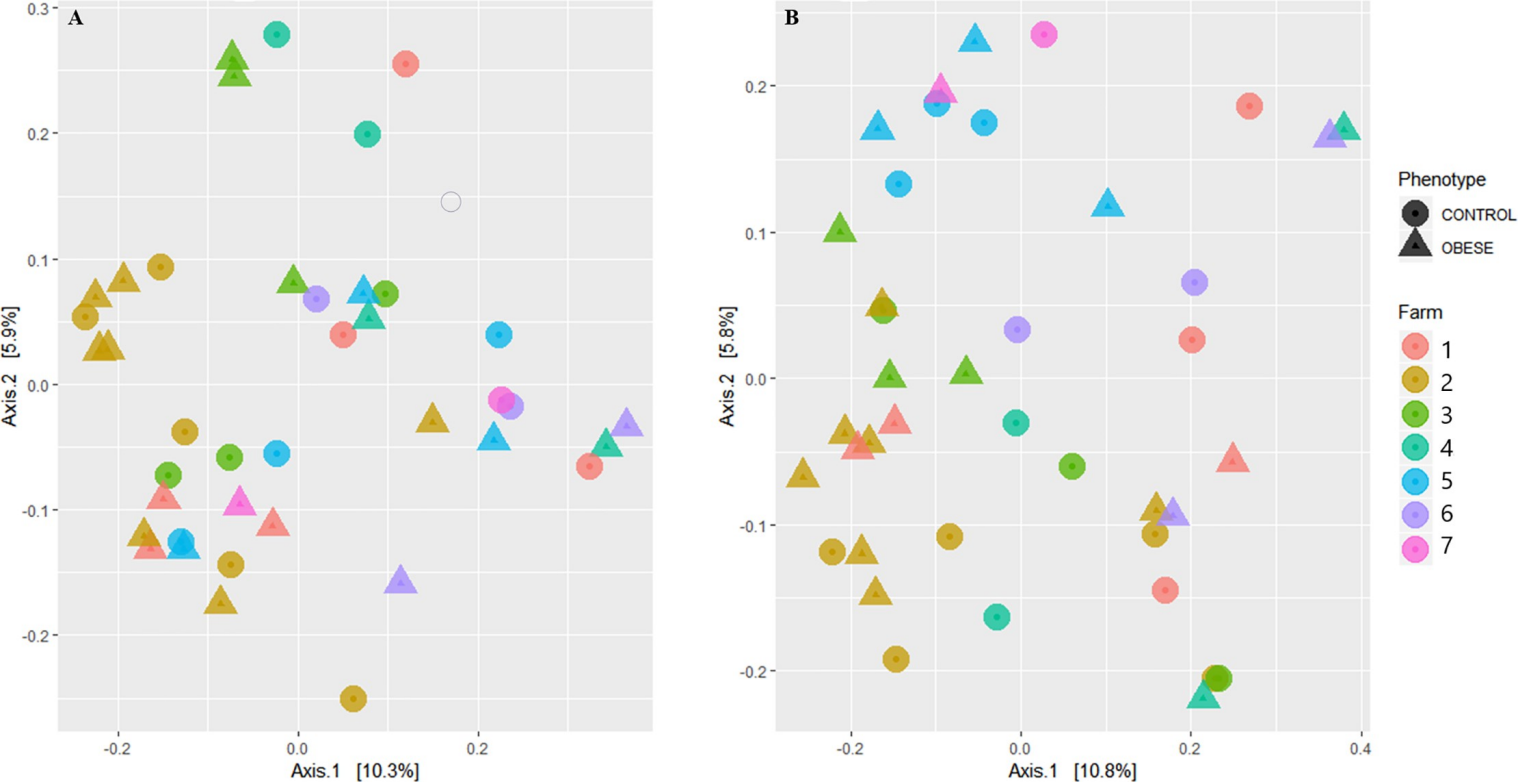
Principal coordinate analysis plots. A) PCoA based on unweighted UniFrac distance metric revealing no visual evidence of clustering by obese (circle) and non-obese(triangle) horses colored by farm B) PCoA based on Bray Curtis distance metric revealing no visual evidence of clustering of obese (circle) and non-obese (triangle) horses colored by farm.

PCoA plots were performed using the unweighted Unifrac distance metric (Fig 2A), which accounts for phylogenetic distance and the Bray Curtis distance metric (Fig 2B) which does not account for phylogenetic distance. Indeed, analysis of similarity (ANOSIM) revealed no clustering based on group (UniFrac: R = 0.007; P = 0.54; Bray: R = 0.0313, P = 0.153).

The EdgeR package was used to examine datasets for differentially expressed OTUs. The Phyloseq object was converted into a matrix of gene counts with taxonomy converted to “genes” for the purpose of analysis. When accounting for only group (case vs. control), there were 8 OTUs differentially expressed (FDR P<0.05), of which only 2 were identified and the remainder were of unknown taxon (S2 Table).

### Fecal metabolomics

A total of 688 biochemicals were evaluated, including 576 compounds of known identity (*i*.*e*., named biochemicals) and 112 compounds of unknown structural identity. These compounds came from the following super-metabolic pathways: amino acid, peptide, energy, lipid, nucleotide, xenobiotics, and cofactors and vitamins. Of the known biochemical, 57 were significantly different (P ≤ 0.05) between obese and non-obese horses when accounting for the effect of farm, with 18 biochemicals higher and 39 lower in cases relative to controls.

**Fig 3:**
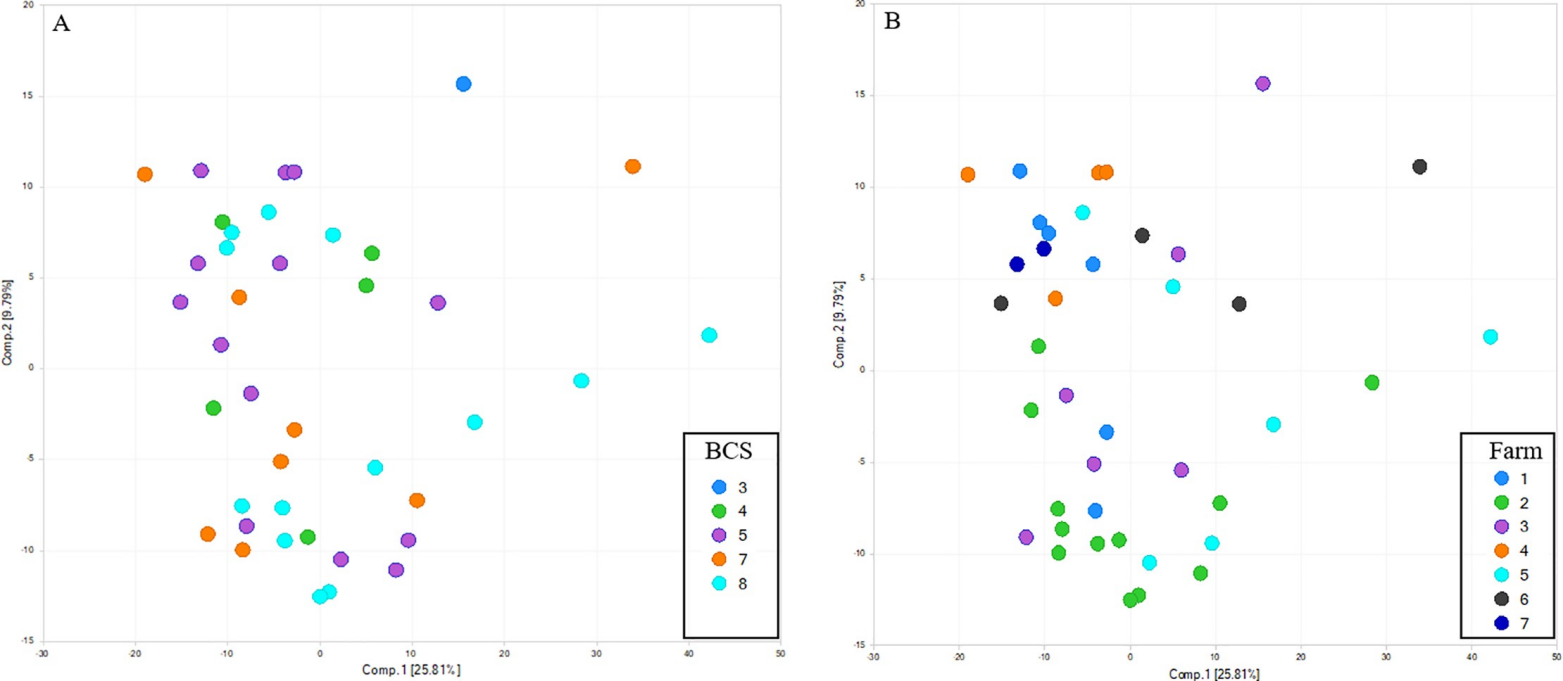
Unsupervised principal coordinate analysis (PCA) of fecal metabolites. A) Unsupervised PCA segregated according to body condition score (BCS) from 3 to 8. Fecal metabolites did not cluster by BCS. B) Unsupervised PCA segregated according based on farm identification (Farm 1–7). Fecal metabolites visually clustered by farm.

Principal coordinate analysis plots demonstrated that, similar to the fecal microbiota, fecal metabolites primarily clustered by farm but not by BCS (Fig 3).

**Fig 4:**
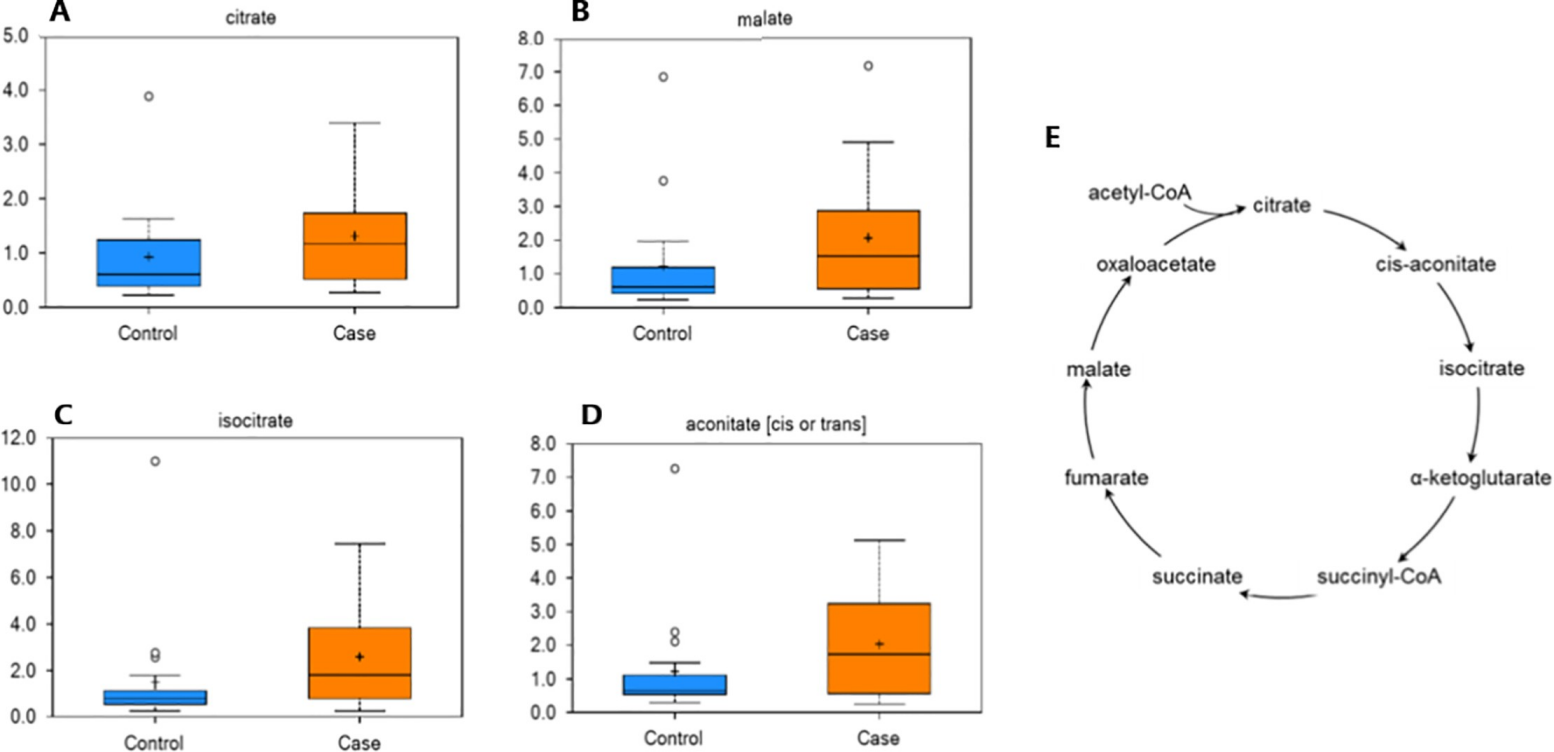
Intermediates of the mitochondrial tricarboxylic acid (TCA) cycle were altered in obese horses. Box plots of the scaled intensity (y-axis) of the TCA cycle intermediates citrate (A), malate (B), isocitrate (C), and aconitate (D) comparing control (blue) and case (orange) (x-axis). The horizontal line of the boxplot is the median, and the top and bottom of the box extend to the 75% and 25% percentiles, respectively. The mean values are indicated by the plus (+) and the filled circles outside the thin horizontal line represent outliers. These TCA cycle intermediates were increased (P≤0.05) in the feces of obese horses. E) A graphical depiction of the TCA cycle is included for reference.

Intermediates of the mitochondrial tricarboxylic acid (TCA) cycle differed between groups, when accounting for the effect of farm. Specifically, isocitrate, malate, citrate, and aconitate were significantly increased in the feces of obese horses (Fig 4).

**Fig 5:**
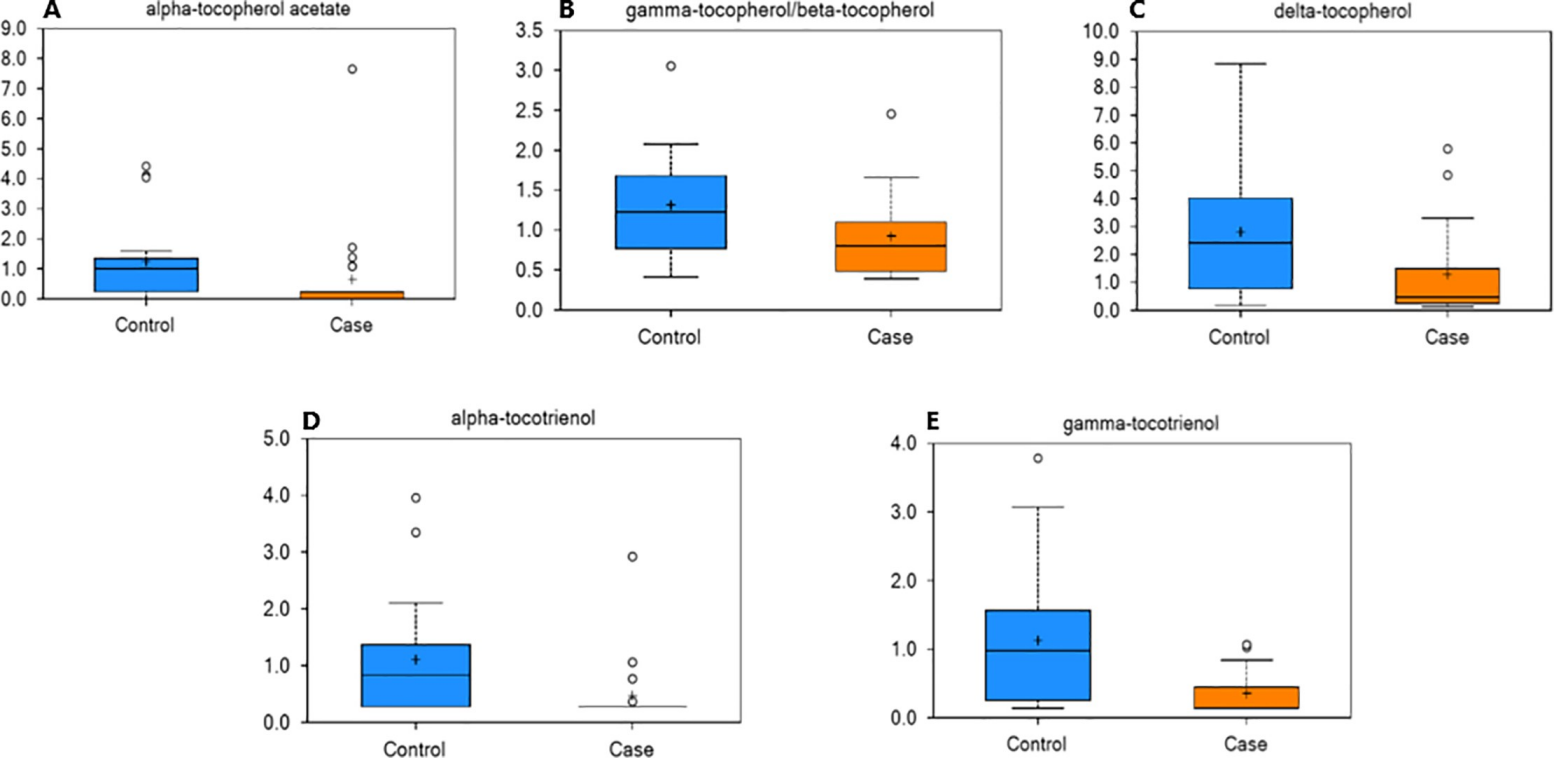
Alterations in Vitamin E of obese horses. Box plots of the scaled intensity (y-axis) of the vitamin E analogs α-tocopherol acetate (A), β-tocopherol (B), delta-tocopherol (C), α-tocotrienol (D), and gamma-tocotrienol (E) comparing control (blue) and case (orange) (x-axis). The horizontal line of the boxplot is the median, and the top and bottom of the box extend to the 75% and 25% percentiles, respectively. The mean values are indicated by the plus (+) and the filled circles outside the thin horizontal line represent outliers. These fecal metabolites were decreased (P≤0.05) in the feces of obese horses.

Tocopherols and tocotrienols were noted to differ between groups. α-tocopherol acetate and tocotrienols, including α-tocopherol acetate, β-tocopherol, delta-tocopherol, α-tocotrienol, and gamma-tocotrienol, were significantly decreased in the feces of obese horses (Fig 5).

### Complex serum lipid profile

**Fig 6:**
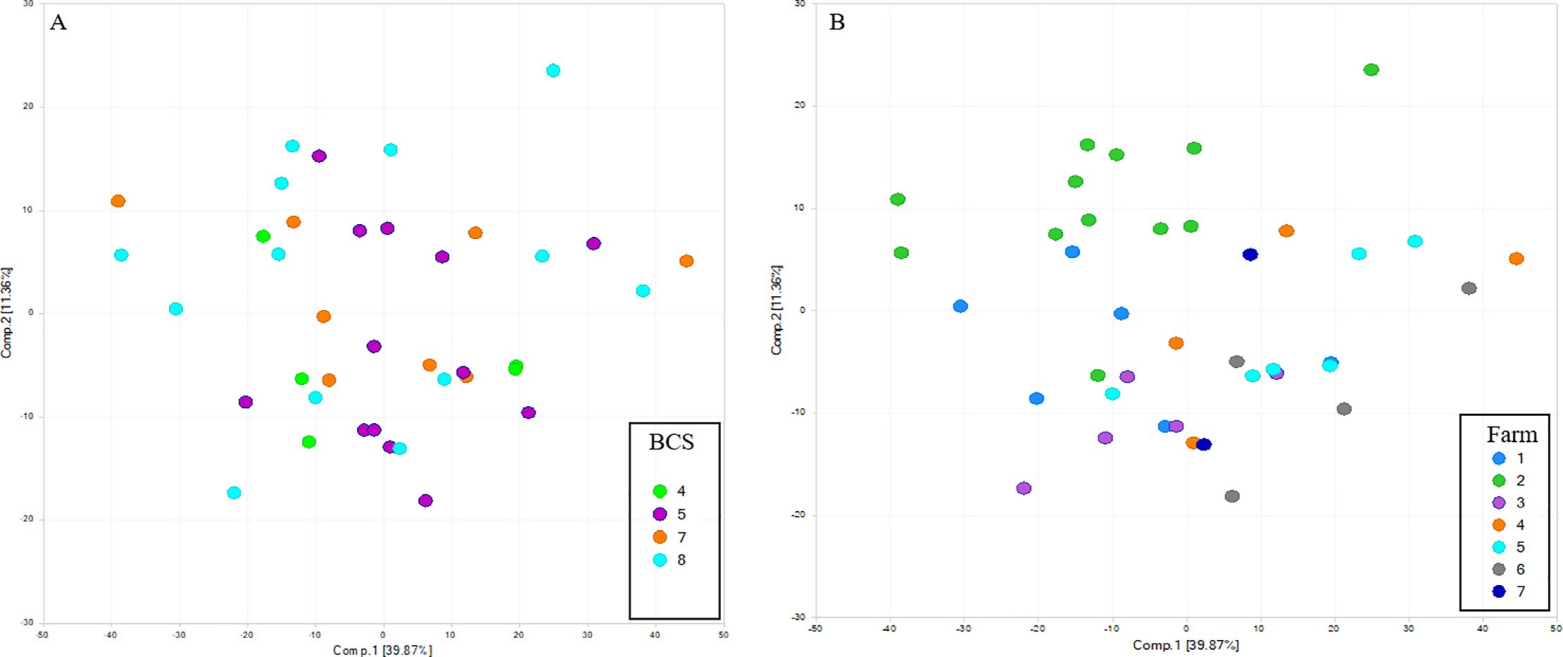
Unsupervised principal coordinate analysis (PCA) of serum lipids. A) Unsupervised PCA segregated according to body condition score (BCS) from 4 to 8, demonstrates no significant visual difference by BCS, B) Unsupervised PCA segregated according based on farm identification (Farm 1–7).

A complex lipid panel (CLP) was performed to quantify the total lipid concentration of 14 different complex lipids including cholesterol esters, ceramides, diacylglycerols, dihydroceramides, free fatty acids, hexosyleramides, lactosylceramides, lysophosphatidylcholines, lysophosphatidylethanolamines, phosphatidylcholines, phosphatidylethanolamines, sphingomyelins, phosphatidylinositols, and triacylglycerols. A total of 936 compounds of known identity were evaluated, of which 146 were significantly (P ≤ 0.05) altered by group, when accounting for the effect of farm. Of these, 110 were significantly higher in cases and 36 were significantly lower in cases, relative to controls. Unsupervised PCA of serum lipids according to BCS (Fig 6A) and farm (Fig 6B), revealed visual differences by farm only.

**Fig 7:**
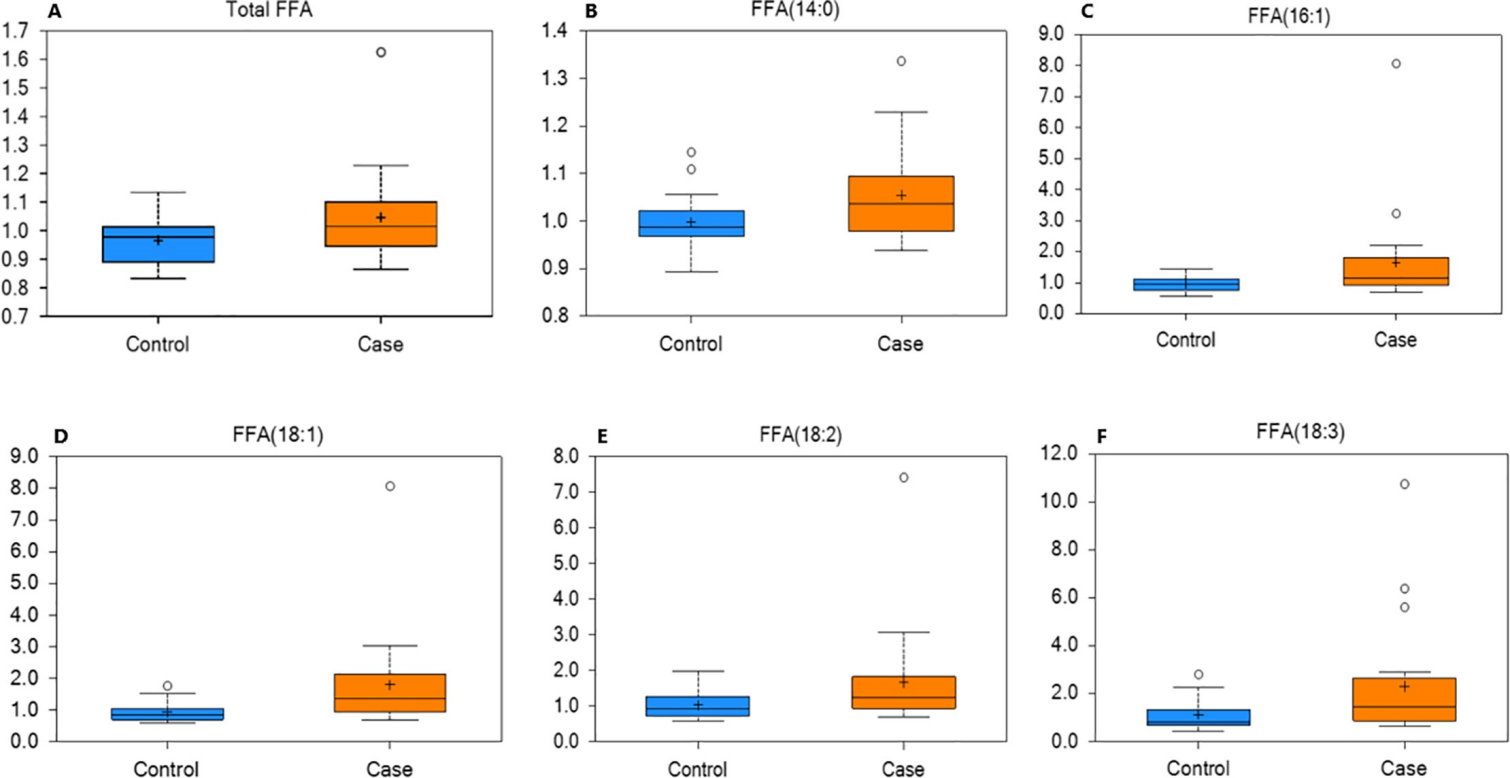
Free fatty acids (FFA) in case and control horses. Box plots of the scaled intensity (y-axis) of total FFA (A) including myristic acid (B), palmitoleic acid (C), oleic acid (D), linoleic acid (E), and α-linoleic acid (F) comparing control (blue) and case (orange) (x-axis). The horizontal line of the boxplot is the median, and the top and bottom of the box extend to the 75% and 25% percentiles, respectively. The mean values are indicated by the plus (+) and the filled circles outside the thin horizontal line represent outliers. These serum fatty acids were increased (P≤0.05) in the serum of obese horses.

Measurement of total lipids in serum revealed a significant increase in total free fatty acids (FFAs) in obese horses relative to controls (Fig 7A). The specific FFAs that were increased in obese horses included myristic acid (14:0), palmitoleic acid (16:1), oleic acid (18:1), linoleic acid (18:2), and α-linoleic acid (18:3) (Fig 7B–7F).

## Discussion

Due to the complexity of bacterial and host interactions in the gastrointestinal tract, a multi-‘omics’ approach is becoming more common in the study of disease. Evaluation of the intersection of the intestinal microbiota and intestinal metabolome is still in its infancy, but it is an emerging field of inquiry using high-throughput molecular analysis platforms. These technologies have enabled considerable progress in providing system-wide insights regarding mechanisms of underlying disease processes in humans, including type 2 diabetes and obesity. These findings have led to identification of biomarkers and risk factors associated with development of disease and novel therapeutic agents for the management and prevention of disease. Despite the relative importance of the gastrointestinal tract and the impact of obesity to the equine population, similar application and progress is lacking for studying obese horses. While the present study did not identify substantial differences between obese and non-obese horses with regard to the GI microbiota, there were important and relevant differences noted in the GI metabolome and circulating lipidome.

The relationship between microbiota and metabolic disturbances has been extensively investigated in several species, including mice and humans. There is mounting evidence in for the role of the gastrointestinal microbiota in body weight and obesity [43, 44], with significant differences in the fecal microbiota demonstrated between lean and obese humans [45, 46] that are suspected to play a role in obesity, IR, and type 2 diabetes in humans.[47] In the present study, there was no significant difference in the alpha richness and diversity indices, nor was there any apparent clustering by group. A total of 8 OTUs were differentially expressed, of which only 2 were identified and the remainder were of unknown taxon. The significance of these findings is unknown. Interestingly, a recent study demonstrated that horses with EMS have differences in their fecal microbiota compared to controls, with EMS horses exhibiting a decreased fecal microbial diversity [48]. In this study, the OTU with the highest linear discriminant analysis effect size in association with EMS was a member of the subdivision 5 of Verrucombicrobia incertae sedis [48]. Verrucomicrobia abundance has been suggested as a microbial biomarker in the progression of glucose intolerance [49]. In the study reported here, however, horses were based solely on phenotype; metabolic status was unknown, suggesting factors such as the role of insulin dysregulation in modulating the gut microbiota may be relevant.

Additionally, while interesting information has been obtained from study of the microbiome, accurate identification of microbes constituting the microbiota can be limited when utilizing sequencing of the conserve 16S ribosomal RNA gene. It has been demonstrated that shotgun whole genome sequencing (WGS) has multiple advantages over 16S amplicon sequencing including enhanced detection of bacterial species, increased detection of diversity and increased prediction of genes [50].

Fecal metabolites reflect the net result of nutrient ingestion, digestion, and absorption by both the gut bacteria and the host GI tract, providing utility to the study of host-microbiota interactions in health and disease. The present study identified significant differences in the fecal metabolome between obese and non-obese horses, specifically with regards to intermediates of the TCA cycle. The TCA cycle is a series of enzyme-catalyzed chemical reactions that drive anaerobic respiration of cells and result in the production of energy in the form of ATP. As these metabolites from the diet are primarily absorbed in the small intestines, it is suspected that fecal TCA cycle intermediates are the result of bacterial metabolism within the GI tract [51], supporting the importance of the microbiome. A decrease in TCA cycle intermediates was observed in rats treated with antimicrobials to suppress the gut microbiome [52]. Further, high-fat diet induced changes in fecal TCA intermediates correlated to changes in the fecal microbiome in rats [53]. These alterations may result in increased energy extraction from dietary nutrients and has been hypothesized as a link between the gut microbiome and obesity [54]. Thus, the results of this study suggest that alterations in energy metabolism of GI bacteria might contribute to the development or persistence of obesity despite an appropriate dietary and exercise regimen. Interestingly, circulating TCA cycle intermediates are decreased in ID ponies and in humans with type II diabetes [55, 56]. The serum metabolome was not investigated in the present study; we plan to follow up on this line of inquiry in the future.

Vitamin E is a family of anti-oxidant vitamins that include tocopherols and tocotrienols. These vitamins are lipid-soluble and have potent antioxidant properties. α-tocopherol acetate and tocotrienols, including α-tocopherol acetate, β-tocopherol, delta-tocopherol, α-tocotrienol, and gamma-tocotrienol, were significantly decreased in the feces of obese horses. The α-tocopherol content of fresh grass is high and declines with storage [57]. Thus, the changes in these compounds might reflect changes in dietary intake, season, or absorption. Studies in rats suggest that between 20% to 80% of α-tocopherol is intestinally absorbed. Vitamin E is not generated by gut bacteria and does not appear to be metabolized by gut bacteria [58]; however, studies in mice suggest that some bacterial species can influence the absorption of vitamin E [59]. Finally, studies in obese humans suggest that chronic, low grade-inflammation induced by obesity may increase vitamin E requirements to combat the associated oxidative stress. As vitamin E levels in feces do not correlate with serum levels, measurements of vitamin E and oxidative stress in serum is warranted.

Advances in lipidomic techniques have provided new insights into the role of lipid metabolism in obesity and obesity-related disorders. In the present study, measurement of total lipids in serum revealed a significant increase in total free fatty acids (FFAs) in obese horses relative to control horses. Free fatty acids are adipose-derived and bound to albumin in blood and absorbed by tissues. These lipids are implicated in the pathogenesis of obesity, with increased levels resulting in insulin resistance, desensitization of adipocytes, and systemic inflammation in humans with metabolic disease and obesity [60, 61].

The lipid profile observed in horses closely resembles the lipid profile of obese humans [62]. The serum concentrations of palmitoleic acid reflects *de novo* hepatic fatty acid synthesis from palmitic acid. Thus, high levels of palmitoleic in the obese group suggests enhanced desaturase activity. High levels of palmitoleic acid have been associated with abdominal obesity, metabolic syndrome, and cardiovascular disease in humans [63]. Increased oleic acid might be explained by an oversupply of fatty acids from excess visceral fat and altered activity of fatty acid desaturase. Linoleic is a precursor of diHOME polyunsaturated fatty acid (PUFA). The PUFAs are thought to promote adipogenesis and increase expression of lipogenic genes. Further, these fatty acids are generated in activated leukocytes and act as neutrophil chemo-attractants. This alteration in the lipid profile of obese horses could be a contributing factor to altered cell signaling and increased inflammation, as is seen in obese humans and mice [64, 65].

Cholesteryl esters (CE), diacylglycerols (DAGs), phosphatidylcholines were increased in the obese horses. These findings also closely resemble the lipidomic alterations in humans with metabolic disease and obesity. The increase in CEs in obese horses was not unexpected given the increase in FFA. Diacylglycerols can be generated through the activity of lipases on triacylglycerides and can be further degraded to monoacylglycerols, with the release of FFA at each step. Finally, many species of phosphatidylcholine were increased in obese horse serum. Phospholipids make up the largest portion of cell membrane lipids and proliferating cells synthesize new phospholipids from diacylglycerols and polar head groups such as choline and ethanolamine. Phosphatidylycholines make up the largest portion of membrane phospholipids. Changes in cell membrane composition alter receptor density and cell signaling have been associated with metabolic syndrome in humans [66]. The changes seen in lipid profiles of the obese horses are similar to those of obese humans [67]. While it is difficult to make direct comparisons between horses and humans, there are several species of DAG, triglycerides and FFAs that have been implicated in human obesity comorbidities, suggesting that these also may be biomarkers of horses at greater risk of developing metabolic disease or as potential therapeutic targets to control for disease development.

Due to the complexity of bacterial and host interactions in the gastrointestinal tract, a multi-‘omics’ approach is becoming more common in the study of disease. Evaluation of the intersection of the intestinal microbiota and intestinal metabolome is still in its infancy, but it is an emerging field of inquiry using high-throughput molecular analysis platforms. These technologies have enabled considerable progress in providing system-wide insights regarding mechanisms of underlying disease processes in humans, including type 2 diabetes and obesity. These findings have led to identification of biomarkers and risk factors associated with development of disease and novel therapeutic agents for the management and prevention of disease. Despite the relative importance of the gastrointestinal tract and the impact of obesity to the equine population, similar application and progress is lacking for studying obese horses.

The results, though interesting and potentially meaningful for identification of biomarkers for obesity, are preliminary and several limitations to the current study exist in which to address the question of whether serum lipidomics, fecal microbiota and metabolomics may provide meaningful data for comparison of horses with an obese phenotype. A cross-sectional study design, which precludes establishing causal relationships between obesity and parameters examined. This study was designed as an initial proof-of-concept study, and substantiation of these observations in larger-scale, prospective studies is needed. Data were obtained from a small and diverse population of horses from several farms. Non-targeted metabolomic studies in human patients with similarly small sample populations have successfully detected biologically important, statistically significant differences [13, 14]; however, it is expected that variability in age, breed, and diet of the horses studied could have played a role in the heterogeneity of microbiotic, metabolomics, and lipidomic profiles. Our imbalanced study design and heterogenous study population including several farms could have diluted our power to detect existing differences between obese and non-obese horses and resulted in a huge amount of variation. Furthermore, diet has been shown to alter the fecal microbiota of horses, and thus likely the fecal metabolites. Differences in the fecal microbiota between forage-only diets and diets which include concentrates have been demonstrated in horses [68, 69]. Obese horses were matched with non-obese horses from the same farm to control for differences in management practices, including dietary management. Thus, the identified effect of farm likely reflects similarities in feeding practices within farms. The clustering of results of farm was controlled in the analysis. Finally, the structure and function of the equine gastrointestinal tract change dramatically from the stomach to the rectum [22]. While fecal samples have been shown to represent the microbiota of the large colon in horses, it is likely that evaluation of feces may only partially reflect the composition of the proximal gastrointestinal tract [70].

Despite these limitations, this study has identified important differences between obese horses in fecal microbiota, fecal metabolome, and serum lipidome. Further investigation utilizing a more homogeneous population is warranted to substantiate the validity of these results and to determine the extent to which the observed alterations could serve as biomarkers for development and control of obesity. More importantly, further investigation and mixomics analysis is warranted to determine whether the observed association between the fecal microbiota and fecal metabolome and the host lipid profile are causally linked, and what cellular mechanisms contribute to this link. Such studies offer the possibility for identifying therapeutic approaches targeting the fecal microbiota and fecal metabolites that could reduce the risk of obesity and related sequelae. Given some of the similarities observed in this study of horses with what is known about humans, the potential exists that information gleaned from studying the relationship of the microbiota, intestinal metabolome, and host metabolism in horses could be relevant to human health.

## Conclusions

In summary, as seen in other species, improved knowledge and understanding of the microbiome and metabolome of obese horses may elucidate information regarding the pathogenesis of disease. The present study identified an increase in fecal TCA cycle intermediates, and increased circulating FFA in obese horses compared to non-obese horses. These findings support important differences in energy metabolism between groups and warrants further investigation. Differences in fecal microbiota were not appreciated; however, further exploration into the metagenome is suggested.

## Supporting information

S1 Table Metadata collected from 20 obese and 20 non-obese horses. Signalment (age, breed, sex), body condition score (1–9), and farm (1–7) of horses included in the study.
(PDF)

S2 Table Differentially expressed OTUs in obese versus non-obese horses. The non-obese group was used as a control group, therefore the negative fold change represents increased abundance of these OTUs in the obese horses.
(PDF)
