# Supplementary material for: Comparison of the microbiome, metabolome, and lipidome of obese and non-obese horses
Source: PLoS One. 2019 Apr 23;14(4):e0215918. doi: 10.1371/journal.pone.0215918 (PMC6478336; doi:10.1371/journal.pone.0215918)
Supplement: S1 Table — Signalment (age, breed, sex), body condition score (1–9), and farm (1–7) of horses included in the study. (PDF) [file pone.0215918.s001.pdf]

Horse	Group	Age (years)	Breed	Sex	Body Condition Score	Farm
1	Obese	7	Quarter Horse	Gelding	8	1
2	Non-Obese	24	Quarter Horse	Gelding	4	1
3	Obese	7	Quarter Horse	Gelding	7	1
4	Non-Obese	21	Thoroughbred	Mare	5	1
5	Obese	14	Warmblood	Gelding	8	1
6	Non-Obese	16	Quarter Horse	Gelding	5	1
7	Obese	16	Quarter Horse	Gelding	7	2
8	Obese	10	Quarter Horse	Mare	8	2
9	Obese	18	Draft	Gelding	8	2
10	Obese	13	Draft	Gelding	8	2
11	Obese	9	Quarter Horse	Gelding	8	2
12	Obese	16	Draft	Gelding	7	2
13	Non-Obese	13	Quarter Horse	Gelding	5	2
14	Non-Obese	14	Quarter Horse	Gelding	5	2
15	Non-Obese	6	Quarter Horse	Gelding	5	2
16	Non-Obese	17	Draft	Mare	4	2
17	Non-Obese	21	Quarter Horse	Gelding	5	2
18	Non-Obese	6	Quarter Horse	Gelding	5	2
19	Non-Obese	14	Quarter Horse	Gelding	3	3
20	Non-Obese	18	Quarter Horse	Gelding	4	3
21	Non-Obese	20	Quarter Horse	Gelding	5	3
22	Obese	18	Quarter Horse	Gelding	7	3
23	Obese	9	Quarter Horse	Gelding	8	3
24	Obese	9	Quarter Horse	Mare	7	3
25	Obese	14	Quarter Horse	Gelding	7	4
26	Non-Obese	5	Quarter Horse	Mare	5	4
27	Obese	17	Quarter Horse	Gelding	7	4
28	Non-Obese	14	Appaloosa	Gelding	5	4
29	Obese	9	Quarter Horse	Gelding	8	5
30	Non-Obese	13	Quarter Horse	Gelding	5	5
31	Obese	14	Quarter Horse	Gelding	8	5
32	Non-Obese	20	Quarter Horse	Gelding	5	5
33	Obese	18	Quarter Horse	Gelding	8	5
34	Non-Obese	6	Thoroughbred	Gelding	4	5
35	Obese	16	Warmblood	Mare	7	6
36	Obese	20	Warmblood	Mare	8	6
37	Non-Obese	20	Warmblood	Mare	5	6
38	Non-Obese	5	Thoroughbred	Gelding	5	6
39	Obese	15	Arabian	Mare	8	7
40	Non-Obese	8	Warmblood	Gelding	5	7
