# Supplementary material for: Comparison of the microbiome, metabolome, and lipidome of obese and non-obese horses
Source: PLoS One. 2019 Apr 23;14(4):e0215918. doi: 10.1371/journal.pone.0215918 (PMC6478336; doi:10.1371/journal.pone.0215918)
Supplement: S2 Table — The non-obese group was used as a control group, therefore the negative fold change represents increased abundance of these OTUs in the obese horses. (PDF) [file pone.0215918.s002.pdf]

Kingdom	Phylum	Class	Order	Family	Genus	Species	log2 fold change	FDR P-value
k__Bacteria	p__Firmicutes	c__Clostridia	o__Clostridiales	f__Ruminococcaceae	g__Ruminococcus	s__	-3.13849	0.0048
k__Bacteria	NA	NA	NA	NA	NA	NA	-3.51583	0.0049
k__Bacteria	NA	NA	NA	NA	NA	NA	-3.07380	0.0087
k__Archaea	p__Euryarchaeota	c__Methanobacteria	o__Methanobacteriales	f__Methanobacteriaceae	g__Methanobrevibacter	s__	-2.93878	0.0221
k__Bacteria	NA	NA	NA	NA	NA	NA	-2.81307	0.0221
k__Bacteria	p__Firmicutes	c__Clostridia	o__Clostridiales	f__Lachnospiraceae	NA	NA	-2.29729	0.0221
k__Bacteria	NA	NA	NA	NA	NA	NA	-1.44473	0.0404
k__Bacteria	p__Bacteroidetes	NA	NA	NA	NA	NA	-2.30031	0.0481
k__Bacteria	p__Bacteroidetes	NA	NA	NA	NA	NA	-1.92803	0.0481
